# A Clinical Perspective on Advanced Developments in Bone Biopsy Assessment in Rare Bone Disorders

**DOI:** 10.3389/fendo.2020.00399

**Published:** 2020-06-23

**Authors:** Sanne Treurniet, Elisabeth M. W. Eekhoff, Felix N. Schmidt, Dimitra Micha, Björn Busse, Nathalie Bravenboer

**Affiliations:** ^1^Department of Internal Medicine, Amsterdam University Medical Center, Amsterdam Movement Sciences, Amsterdam, Netherlands; ^2^Department of Osteology and Biomechanics, University Medical Center Hamburg-Eppendorf, Hamburg, Germany; ^3^Department of Clinical Genetics, Amsterdam University Medical Center, Amsterdam Movement Sciences, Vrije Universiteit Amsterdam, Amsterdam, Netherlands; ^4^Bone and Calcium Metabolism Lab, Department of Clinical Chemistry, Amsterdam University Medical Center, Amsterdam Movement Sciences, Amsterdam, Netherlands

**Keywords:** bone biopsy, histomorphometry, bone quality, rare bone disorders, advanced techniques

## Abstract

**Introduction:** Bone biopsies have been obtained for many centuries and are one of the oldest known medical procedures in history. Despite the introduction of new noninvasive radiographic imaging techniques and genetic analyses, bone biopsies are still valuable in the diagnosis of bone diseases. Advanced techniques for the assessment of bone quality in bone biopsies, which have emerged during the last decades, allows in-depth tissue analyses beyond structural changes visible in bone histology. In this review, we give an overview of the application and advantages of the advanced techniques for the analysis of bone biopsies in the clinical setting of various rare metabolic bone diseases.

**Method:** A systematic literature search on rare metabolic bone diseases and analyzing techniques of bone biopsies was performed in PubMed up to 2019 week 34.

**Results:** Advanced techniques for the analysis of bone biopsies were described for rare metabolic bone disorders including Paget's disease of bone, osteogenesis imperfecta, fibrous dysplasia, Fibrodysplasia ossificans progressiva, *PLS3* X-linked osteoporosis, Loeys-Diets syndrome, osteopetrosis, Erdheim-Chester disease, and Cherubism. A variety of advanced available analytical techniques were identified that may help to provide additional detail on cellular, structural, and compositional characteristics in rare bone diseases complementing classical histopathology.

**Discussion:** To date, these techniques have only been used in research and not in daily clinical practice. Clinical application of bone quality assessment techniques depends upon several aspects such as availability of the technique in hospitals, the existence of reference data, and a cooperative network of researchers and clinicians. The evaluation of rare metabolic bone disorders requires a repertoire of different methods, owing to their distinct bone tissue characteristics. The broader use of bone material obtained from biopsies could provide much more information about pathophysiology or treatment options and establish bone biopsies as a valuable tool in rare metabolic bone diseases.

## Introduction

Trepanning of bone is one of the oldest medical procedures known in history. This procedure was initially carried out for the treatment of headache and mental illness. The first application of trepanning as a diagnostic tool was described early 1900 by Pianese for bone marrow aspiration. During the last century, different needles have been developed, including a type for bone biopsy acquisition (1). The first modern needle for the performance of an iliac crest biopsy was described in 1954 (2). Around 1950, modern embedding techniques were discovered for the microscopic examination of mineralized bone tissue (3). Beside conventional X-rays and biochemical markers, histopathological analyses were considered for many years to be the hallmark for the diagnosis of bone-related disorders. The publication of standardization of histomorphometric nomenclature in 1987, largely improved communication between practitioners of bone histomorphometry, medical doctors, and scientists (4). This led to a broader understanding of histomorphometric data (5). The introduction of more advanced radiographic imaging techniques has complemented the role of bone biopsies are no longer the only diagnostic tool for bone disorders. Dual energy x-ray absorptiometry (DXA) is a widely used method to measure bone mineral density to estimate fracture risk. However, measurement of bone mineral density does neither provide information about the microarchitecture of bone, bone cell activity, and bone remodeling, nor allows the consideration of bone volume and density independently of each other. Histology and quantitative histomorphometric analyses are still the most commonly used method for analyzing bone samples; this is often combined with double-labeling with tetracycline which provides additional information about bone mineralization.

The indication for a bone biopsy has shifted from diagnostics of bone structural changes to an additional assessment of compositional information at the tissue level. This shift has taken place due to the availability of new imaging and analyses techniques, a broader use of biochemical markers, and genetic testing. Nowadays bone biopsies are indicated in patients with early onset osteoporosis, renal osteodystrophy, malignant disorders, suspicion of osteomalacia, or rare (metabolic) bone disorders for the analysis of bone mass, cortical and trabecular structure, bone turnover and mineralization as well as cellular status. All these parameters need to be considered in the thorough evaluation of bone quality. Here, bone quality can be considered as the total sum of characteristics with the ability to resist bone fracture, in particular the combination of structural and compositional bone characteristics, as well as the bone cellular activity. Impaired bone quality or bone fragility leads to fractures. Certainly, bone histomorphometric assessment in addition to *T*-Scores measured with DXA remains useful to predict fracture risk in different rare metabolic bone disorders. However, specific techniques for analyzing bone quality in bone biopsies have become available to examine the pathophysiology in rare bone diseases in more detail. These additional analysis techniques include Fourier transform infrared spectroscopy (FTIR), Raman spectroscopy, transmission electron microscopy (TEM), quantitative backscattered electron imaging (qBEI), confocal microscopy, microCT (μCT), micro- and nano-indentation, high-performance liquid chromatography (HPLC), atomic force microscopy (AFM), small-angle X-ray scattering (SAXS), wide-angle X-ray scattering (WAXS), nuclear magnetic resonance (NMR), and immunohistochemistry (Table 1). Currently, the aforementioned techniques are predominantly applied in research settings and are mostly not used in routine clinical practice. This review focuses on the state-of-the-art assessment of bone biopsies from rare bone diseases. Furthermore, additional information on comprehensive bone quality analyses and its impact for clinical practice is given as a perspective. Such techniques allowing for high-resolution analyses of osseous changes in combination with traditional bone histomorphometry represent a valued resource for the diagnosis of rare metabolic bone disorders and the refined prediction of bone fragility.

**Table 1:** Overview of different techniques which can be considered in bone biopsy analysis by clinicians and researchers.

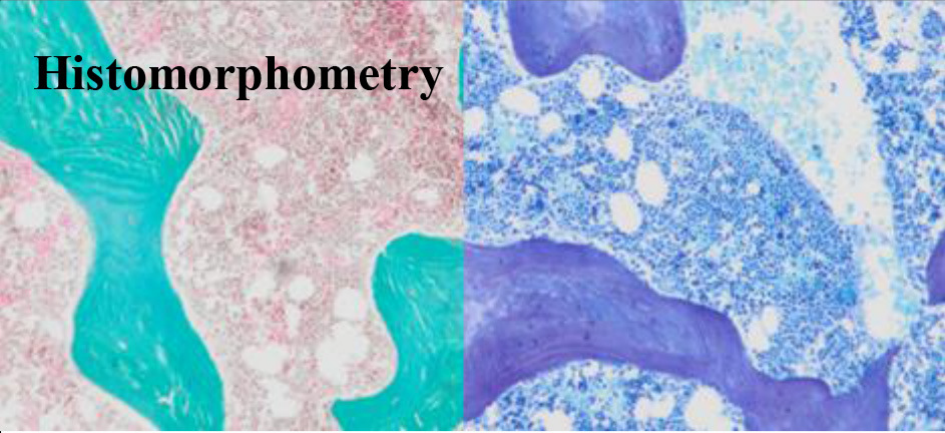	Histomorphometry is the gold standard for the assessment of bone cell activity at the tissue level. It can be used for static quantitative histomorphometric analysis of bone mass, bone structure, bone turnover and dynamic mineralization kinetics after tetracycling labeling. It can also be used to monitor treatment. Standardization of parameters for quantification are published by Dempster et al. (5)
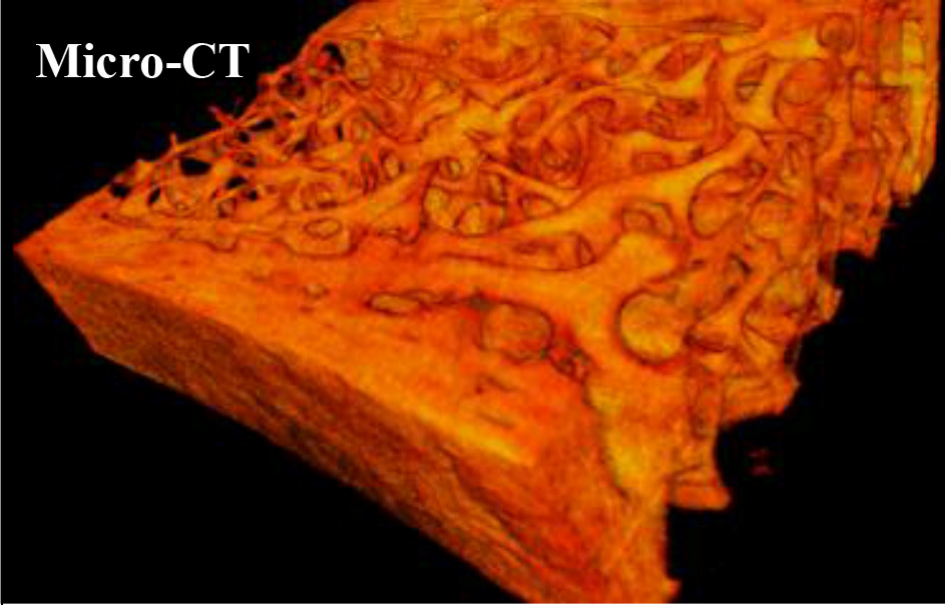	Microcomputed tomography is a technique to investigate cortical and trabecular bone morphology. Only mineralized bone tissue can be imaged. Individual slices can be combined to produce a 3-dimensional reconstruction of the sample. Non-destructive, intact bone samples from biopsy can be scanned in spatial resolutions ranging from 1 to 30 μm. Structural analysis can be carried out on the 3D information/model. To virtually investigate the mechanical properties of the bone, finite element analysis can be applied to the three-dimensional model. Thus, structural features as well as mineralization and its influence on the mechanical performance of bone can be estimated
**Microradiography**	Quantitative X-ray imaging of an unstained section of a bone biopsy. The radio-opaque mineralized bone present in a given field of tissue area can be selectively measured by this technique and provides detailed information on the bone mineral density with high spatial resolution (6)
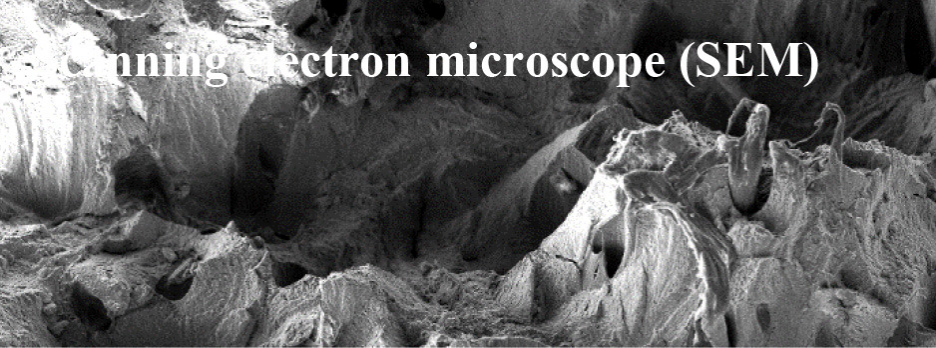	The scanning electron microscope (SEM) scans a focused electron beam over a surface. The electrons in the beam are interacting with the sample, which results in signals that reflect information on the samples surface topography. The electrons that are reflected off the samples surface region are then used to form an image. Thus, secondary electrons are most valuable for showing morphology and topography aspects of the samples
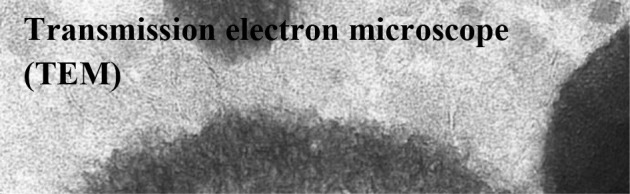	Electrons are submitted through a thin layer of tissue. Transmitted electrons are detected and represent an image of electron absorption of the tissue lamella, thus the special density of the lamella. This technique visualizes nanometer-sized structures of collagen, mineral, and cellular organelle/features (7, 8)
**Confocal microscopy**	Confocal microscopy is an optical imaging technique with high optical resolution and contrast of a micrograph by means of using a spatial pinhole to block out-of-focus light in image formation. Capturing multiple two-dimensional images at different depths in a sample creating a reconstruction of three-dimensional structures at a subcellular level
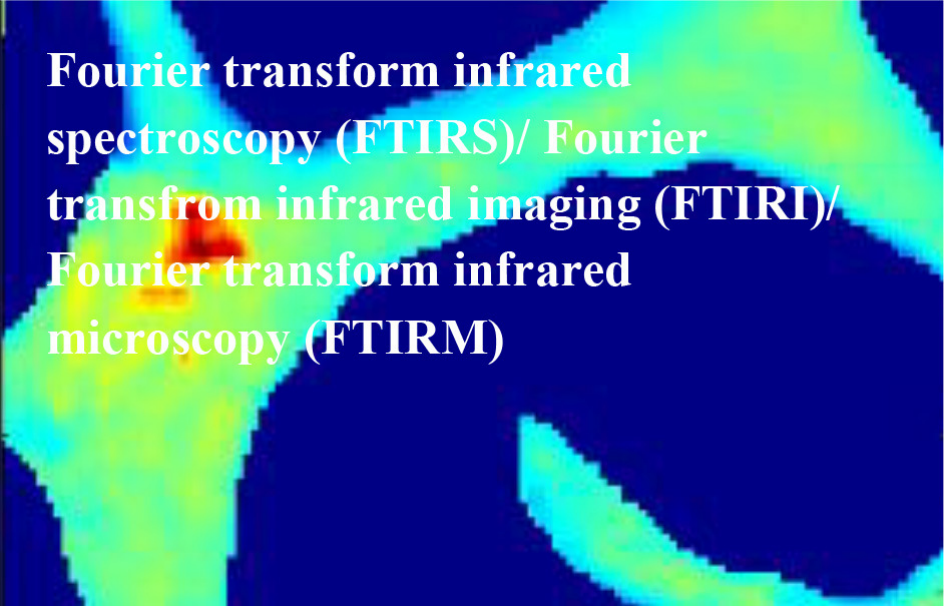	FTIRS is a vibrational spectroscopy technique to analyze bone and tissue material composition (i.e., mineral and collagen). Bone samples are investigated with infrared light which is absorbed by molecule vibrations. The different patterns of absorption distinguish between different molecules of bone material representing the mineral phase and collagenous phase (9, 10). Primarily used outcome parameters of FTIRS are: mineral-to-matrix ratio (ratio of collagen and hydroxyapatite), mineral maturity and collagen-/matrix- maturity and collagen cross-links (11, 12). FTIRS enables representative large-scale mappings/measurements with high spatial resolution in a short time
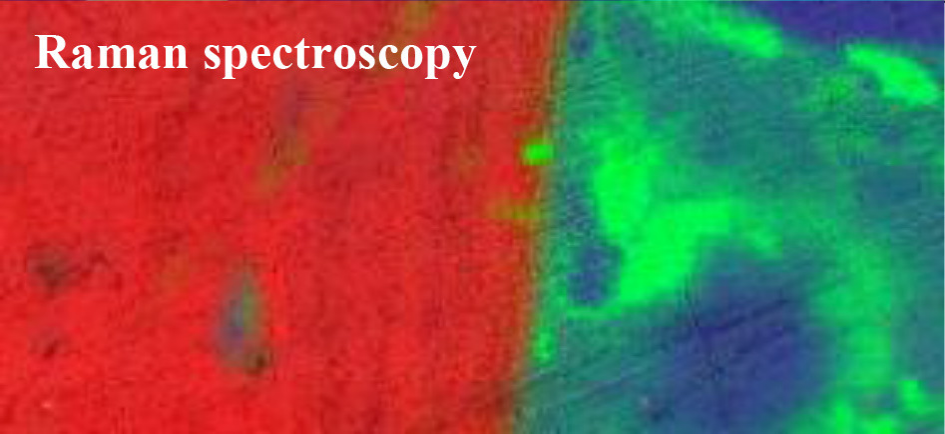	Raman spectroscopy is another vibrational spectroscopy technique. In Raman spectroscopy scattered photons are used to quantify the molecular composition of the tissue. This method, as FTIRS, enables a quantitative assessment of the collagen and mineral phase of bone. Chemical/compositional properties of the tissue can be described by means of mineral-to-matrix ratio, bone mineral crystallinity, collagen quality, and collagen cross-linking (13)
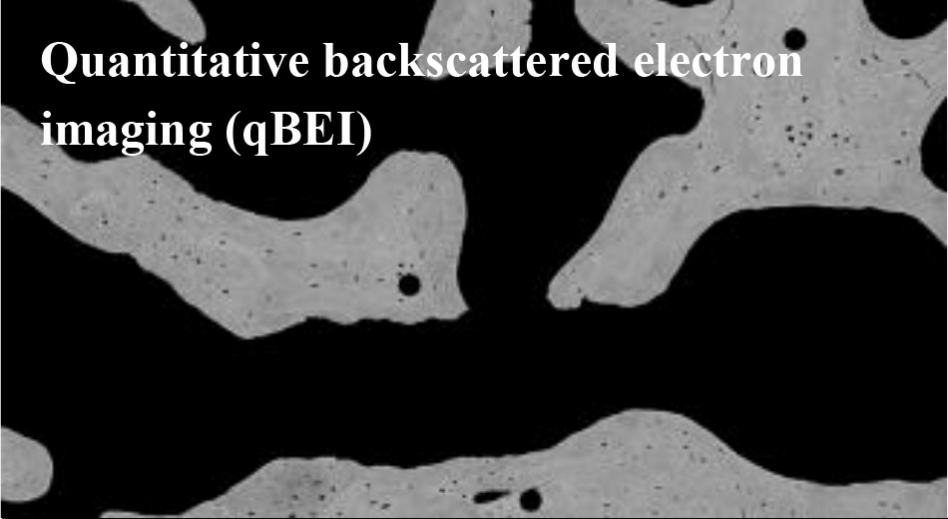	High resolution technique to visualize bone matrix mineralization. Electrons are emitted to a plane surface of embedded bone. The number of backscattered electrons correlates with the mineral density of the tissue. More precisely, the calcium content represents the amount of mineral in this method. The spatial resolution of this method enables to quantify the bone mineral density distribution (BMDD). Due to a calibration to known materials and its concentration the gray values represent a certain amount of mineral, thus this method is quantitative (14)
**High-performance liquid chromatography (HPLC)**	HPLC is used to quantify the amount of molecules in bone. A separation can be achieved by size, charging and others. This method is mainly used to quantify the collagen of bone and, e.g., its cross-linking (10, 15)
**X-ray cristallography**	X-ray cristallography utilizes synchrotron radiation in different scattering/diffraction angles. Parallel X-rays get scattered by periodic, crystalline structures. The scattering/diffraction pattern represent the inner structure of the examined material. Here the diffraction of X-rays represents not only the structure of mineral particles by means of length and thickness. Collagen and mineral alignment can be measured and the collagen-mineral interaction under loading can be determined (16, 17). X-ray crystallography can be divided into SAXS (small-angle X-ray scattering) and WAXD (wide angle X-ray diffraction)
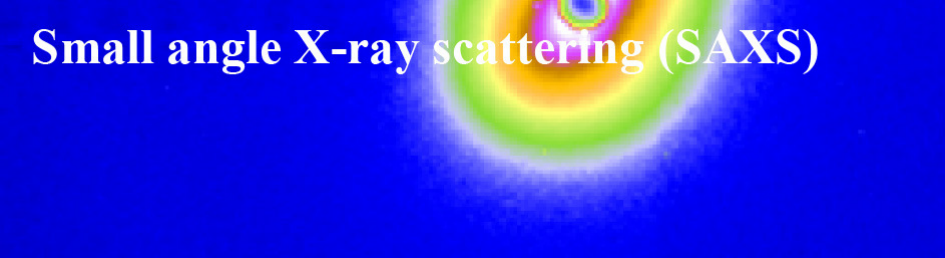	The small angle X-ray scattering can be used to quantify nanoscale density differences. In bone samples, it can be used to analyze ultrastructural orientation and measurement of the size of mineral crystals and collagen arrangement (18, 19). Specifically, particle size and its orientation can be determined (20)
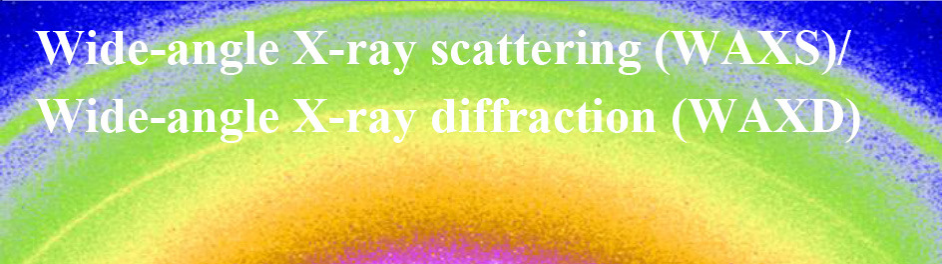	(WAXS)/WAXD works by similar principles like SAXS, however the distance from the sample to the collector is shorter, thus it records the wide angle diffracted x-ray signals. This method can be used in bone to investigate the crystal lattice and the size of hydroxyapatite crystals (19, 20)
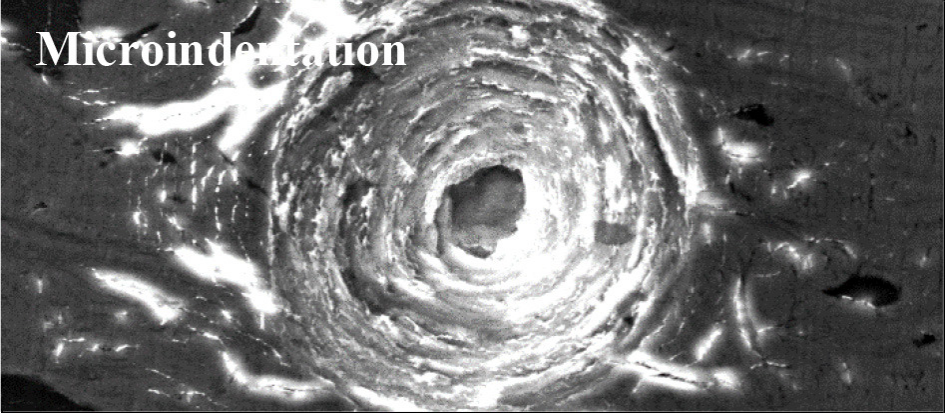	Microindentation is a technique to measure the local biomechanical characteristics of a sample. Here, not a single lamella of bone can be indented but a cluster of neighboring bone lamella, thus it represents a more averaged mechanical characterization on a larger micro-scale
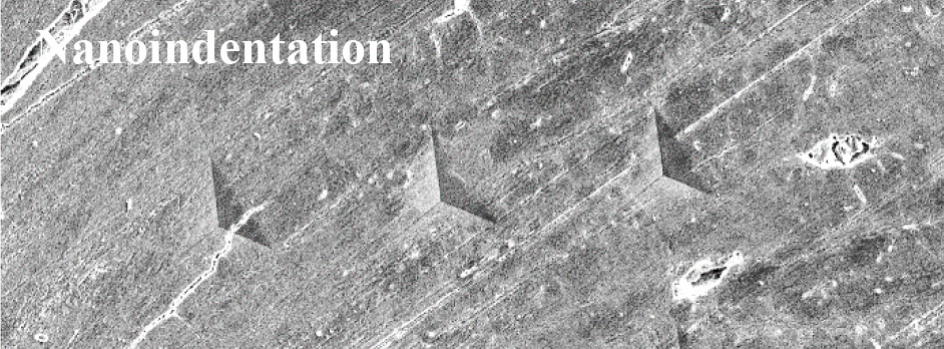	Nanoindentation is a technique to measure the hardness and Youngs's Modulus of small volumes of material. Small indentations are made while measuring the loads and displacements of the indenter. Because of the nanoscale, mechanical properties of different parts of the bone—like individual trabeculae and interstitial lamellae—can be analyzed. This technique is very sensitive for (sub)surface porosity
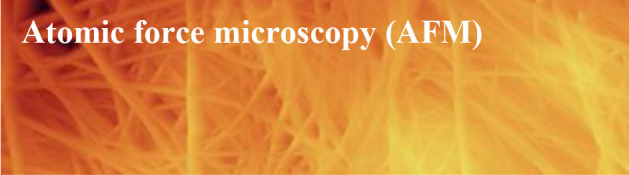	AFM could perform surface measurements on the nanoscale. With this technique, measurement of single collagen fibers and crystal size can be made. AFM has two function abilities: force measurement and topographic imaging (21)
**Immunofluoresence microscopy/Immunohistochemistry**	Specific antibodies bind to specific proteins, and are visualized, resulting in localization at the tissue level. These proteins can be visualized using fluorescence microscopy or light microscopy. 3-dimensional imaging of fluorescent labeled proteins is done by confocal laser microscopy. Quantification is possible with image analysis
**Nuclear magnetic resonance (NMR)**	NMR is a spectroscopic method to measure compositional aspects of bone. NMR uses the nuclear magnetic resonance after sample excitation by radio waves in a magnetic field. It can be used to quantify water content and mineral structure of bone biopsy specimens as well as changes in the mineral chemistry (22)

Therefore, the aim of this review is to give an overview of various rare metabolic bone diseases and the possible use of advanced techniques to analyze bone quality in bone biopsies in the clinical setting.

## Methods

### Literature Search

Literature was systematically reviewed to identify publications on rare metabolic bone diseases and analyzing techniques for bone biopsies. The literature search was performed based on the Preferred Reporting Items for Systematic Reviews and Meta-Analyses (PRISMA)-statement. To identify all relevant publications a systematic search was conducted in the US National Library of Medicine National Institutes of Health (PubMed) for publications from inception to 2019 week 34. The following search terms were used: “*(PLS3 mutation)* OR *(osteogenesis imperfecta)* OR *(osteopetrosis)* OR *(fibrodysplasia ossificans progressiva)* OR *(myositis ossificans)* OR *(osteosclerosis)* OR *(osteomalacia)* OR *(Ehlers-danlos syndrome)* OR *(fibrous dysplasia of bone)* OR *(fibrous dysplasia)* OR *(osteitis deformans)* OR *(paget disease)* OR *(morbus paget)* OR *(m. paget)* OR *(Loeys-dietz syndrome)*” AND “*(histology)* OR *(histochemistry)* OR *(histomorphometry)* OR *(Fourier transform infrared spectroscopy)* OR *(Fourier transform infrared imaging)* OR *(Raman spectroscopy)* OR *(Transmission electron microscopy)* OR *(Quantitative backscattered electron imaging)* OR *(Confocal microscopy)* OR *(Micro-CT)* OR *(contrast enhanced microCT)* OR *(Microindentation)* OR *(Nanoindentation)* OR *(High-performance liquid chromatography)* OR *(Atomic force microscopy)* OR *(Immunofluoresence microscopy)* OR *(Small-angle X-ray scattering)* OR *(Wide-angle X-ray scattering)* OR *(synchrotron)* OR *(*“*NMR*”*)* OR *(*“*Nuclear magnetic resonance*”*)*.”

### Inclusion Criteria

The following inclusion criteria were used: (1) studies describing bone biopsies in the following rare metabolic bone disorders: Paget's disease of bone, osteogenesis imperfecta, fibrous dysplasia, fibrodysplasia ossificans progressiva, *PLS3* X-linked osteoporosis, Ehlers-Danlos syndrome, Loeys-Diets syndrome, osteopetrosis; (2) study published as an original article; (3) adequate description of bone biopsy analysis; (4) studies were published in either English or Dutch; (5) full text availability; and (6) all types of study design.

### Exclusion Criteria

The following exclusion criteria were uses: (1) studies focusing on animal models and (2) studies focusing on *in vitro* models.

### Study Selection

**Figure 1:**
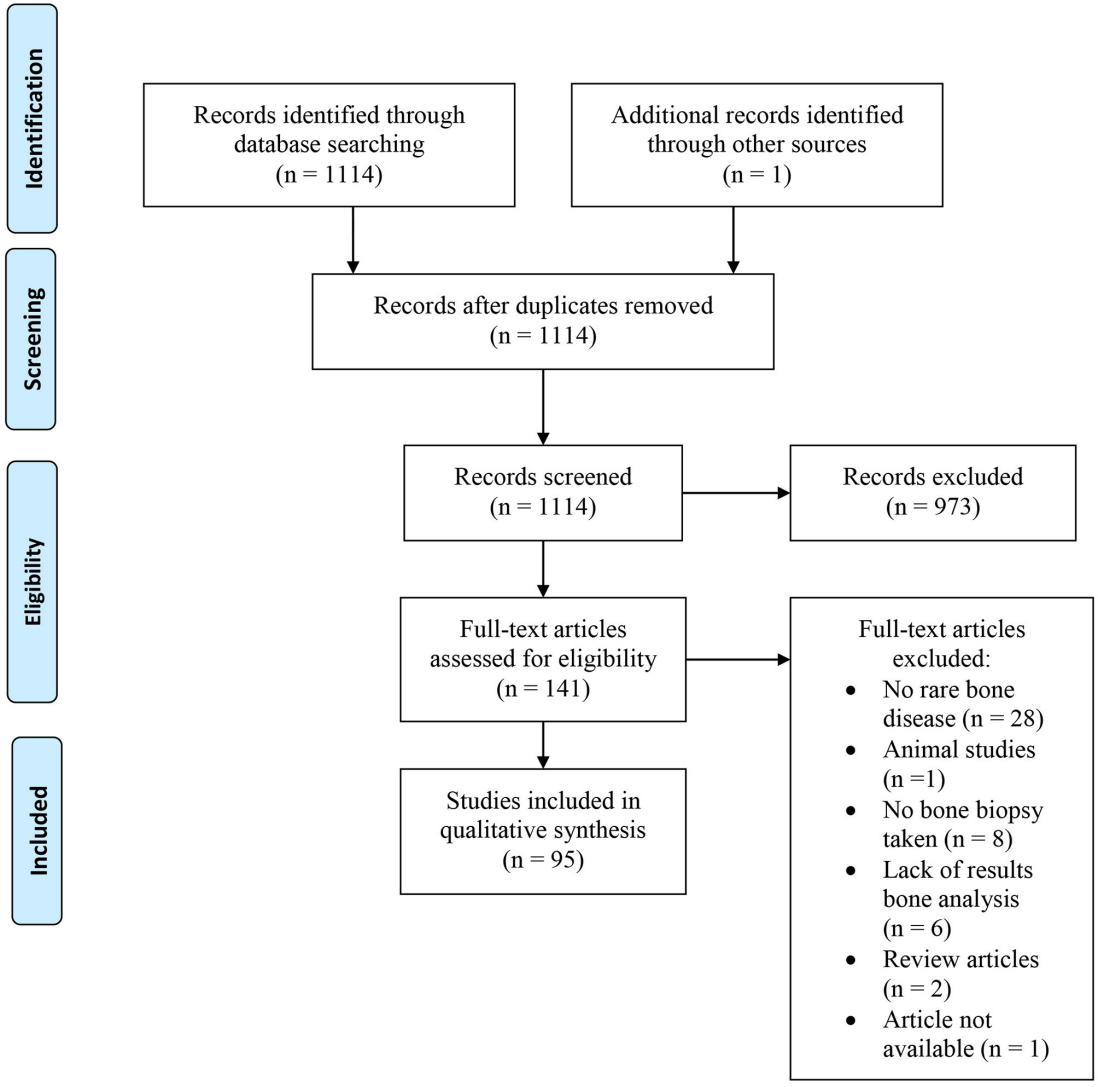
Flow chart of the study selection process.

A total of 1,114 papers were identified. All titles and thereafter remaining abstracts were screened for eligibility by two clinical researchers, ST and EE. In case of disagreement consensus was reached by dialogue. Articles were included when they described the analyzing techniques of bone biopsies in the listed rare metabolic bone disorders. A total of 141 studies were included for initial full text analysis. Forty-six studies were excluded when they did not report any results of the analyzed bone biopsies, described no bone biopsies in any of the listed rare metabolic bone diseases, or were used for *in vitro* studies (Figure 1).

### Data Extraction

The extracted data included the following: (1) name of the first author; (2) year of publication; (3) journal; (4) number of patients with a bone biopsy; (5) aim of the article; (6) type of material (biopsy or residual); (7) preparation method; (8) technique of bone material analysis; (9) results.

### Study Quality Assessment

Study quality assessment was performed on all articles. The quality assessment was independently conducted by two clinical researchers, ST and EE. In case of disagreement consensus was reached by dialogue. Thirty-two (34%) articles consisted of case reports. The remaining 63 articles were assessed using the Study Quality Assessment Tools created by the NHLBI (23). Two items were added to the tool: “Were the techniques used for analysis of bone biopsy material well-described?” and “Were the histomorphometric parameters well-described?”

## Results

A final total of 95 studies were included in this review. Results of the quality assessment (NHLBI) of included studies showed that 10% of the studies were classified as poor, 73% as fair and 17% as good (23). An overview of the included studies and quality assessment is displayed as a supplement. Table 2 shows an overview of the different analysis techniques per rare metabolic disorder as found in the PubMed search.

**Table 2:** Number of articles describing different analyzing techniques of bone biopsies.

		**Paget**	**OI**	**FD**	**FOP**	**PLS-3**	**LD**	**Osteopetrosis**	**EC**	**Cherubism**
	Number or articles	19	45	9	3	6	1	6	3	3
Structural properties	Histomorphometry	14	27	8	2	6	1	5	1	3
	Micro-CT	1	4							
	Microradiography		1							
	Scanning electron microscope		2							
	Transmission electron microscopy	2	3					4		
	Electron microscopy		2							
	Confocal microscopy			2		1				
Material properties	Fourier transform infrared spectroscopy	1	3		1			1		
	Raman spectroscopy		3			1				
	Quantitative backscattered electron imaging	1	10	1		2	1			
	High-performance liquid chromatography		1							
	X-ray diffraction		1					1		
	Small-angle X-ray scattering	1	1							
	Wide-angle X-ray scattering	1	1							
Mechanical properties	Microindentation	1								
	Nanoindentation		6							
	Three point bending test		2							
	*In situ* fracture Thoughness test	1								
	Vickers-hardness							1		
Immunology	Immunofluoresence microscopy	1								
	Immunohistochemistry	1	3	4	1	1		1	3	2
	Immunocytochemistry	2								
	Histochemistry		1							
	Nuclear magnetic resonance		1							

*Paget, Paget disease; OI, Osteogeneis imperfecta; FD, fibrous dysplasia; FOP, fybrodysplasia ossificans progressive; PLS3, PLS3 X-linked osteoporosis; LD, Loeys-Dietz syndrome; EC, Erdheim Chester disease*.

Atomic force microscopy was not previously described in any of the rare metabolic bone diseases. There were also no studies found describing bone biopsies in Ehlers-Danlos syndrome. Some studies described other methods than the ones used in our PubMed search: microradiography, scanning electron microscopy, electron microscopy, X-ray diffraction, three-point-bending test, *in situ* fracture toughness test, immunohistochemistry, immunocytochemistry, and histochemistry. These articles were included in this review as well. In addition, Erdheim-Chester disease and Cherubism were included in the results.

### Paget's Disease of Bone

Paget's disease is a chronic focal bone disorder of unknown cause, most commonly seen in elderly patients. The hallmark of this disorder is locally increased bone turnover with uncontrolled osteoclast activity, leading to the resorption of bone at specific skeletal sites. As a result of bone resorption and subsequent increase of osteoblast activity, overproduction of newly formed bone takes place. This newly formed bone is disorganized and has a different tissue composition in comparison to bone regions unaffected by Paget's disease of bone (24). Commonly affected areas are the skull, spine, pelvis, and long bones of the lower extremity. Most patients are asymptomatic; however, some patients suffer from pain and fracture risk can be increased. The diagnosis is primarily based on radiologic evaluation of lytic lesions or thickened cortices, accentuated trabeculae, and increased size of bones or by skeletal scintigraphy (25). Biochemical screening shows an elevated serum concentration of alkaline phosphatase in most patients.

Histological examination of lesions shows excessive numbers of multinucleated osteoclasts and abnormal deposition of primarily woven bone. The bone marrow contains an increased number of blood vessels and bone precursor cells (26).

Nineteen articles on Paget's disease were identified with the PubMed search (24, 27–44). Multiple articles described different immunological techniques in search of a viral origin in the disorder. Most of the articles were published before the year 2000. This may be the reason for which most articles mainly describe histomorphometric data. The study of Zimmermann et al. applied different techniques to characterize the composition, structure, and mechanical properties of Pagetic bone. Quantitative backscattered electron microscopy showed a lower degree of mineralization in agreement with the mineral-to-matrix ratio measured by Fourier transform infrared spectroscopy. Structural properties were analyzed by micro-CT, which showed replacement of the typical longitudinal-oriented Haversian canals by disorganized clusters of bone with high porosity. Also, the trabecular thickness was shown to be substantially higher in Pagetic bone. Mechanical properties were tested by nano-indentation, reference point indentation (micro-indentation), and *in situ* fracture toughness test. These measurements demonstrated a lower plasticity in Pagetic bone (24). Giannini et al. used SAXS and WAXS techniques to characterize the organization of mineral nanocrystals in one patient with Paget's disease. Results showed a disorganized structure of the nanocrystals (44).

### Osteogenesis Imperfecta

Osteogenesis imperfecta (OI) is an inherited disorder which is mainly characterized by increased risk of fractures, blue sclerae, dentinogenesis imperfect, and hearing loss. OI is most commonly caused by mutations in the *COL1A1* or *COL1A2* genes (coding for type I collagen molecules). Type I collagen is the main structural protein in bone, building the mineralizing matrix. OI is subdivided in types I–V, depending on the type and severity of clinical symptoms (45). The production of type I collagen by osteoblasts is abnormal or decreased in patients with OI. In addition to the altered structure, there is hypermineralization of the bone matrix, independent of the type and severity of the disease (46, 47). Histological examination showed low cancellous bone volume. Lamellae appear thinner and less smooth than in healthy people. Histological distinction between OI and other osteoporotic bone disorders by histomorphometric analysis is difficult (48, 49). The diagnosis of OI is based on the described clinical characteristics, radiological findings, and genetic testing.

OI is the most commonly studied rare metabolic bone disorder in our PubMed search with a total of 45 included articles (48, 50–92). This is also the disorder in which high-resolution techniques were employed for in-depth analyses. In particular, quantitative backscattered electron imaging (qBEI), and nanoindentation have been used in multiple studies. Bone mineral density distribution measured by qBEI was increased in different types of OI (58, 59, 61, 62, 68, 69, 76, 83, 90). Only the article by Webb et al. described several OI type XIV patients in which qBEI showed a normal bone mineral density distribution, with the exception of one patient with a higher BMDD compared to healthy controls. This patient had a very low bone turnover (82). In accordance to the high BMDD values found with qBEI, Fratzl-Zelman et al. studied the mineral particle size in children with OI type I with SAXS/WAXD technique. They concluded that high BMDD in children with OI type I is due to increased particle packing density and not to increased particle size (90). Nanoindentation showed a lower Young's modulus in OI in comparison to control bone (50, 53). Comparison between different clinical types of OI was studied. Albert et al. described a higher elastic modulus and hardness in OI type I compared to OI type III (51). Fan et al. showed no significant difference in Young's modulus and hardness between OI type III and OI type IV (52). The data of Weber et al. showed no effect of pamidronate treatment on elastic modulus and hardness (83). In the preliminary study by Abidin et al., the researchers used synchrotron tomography to characterize the 3D structure of cortical bone in children with OI. With these cortical characteristics they successfully performed a machine learning task to distinguish between OI and healthy bone. They conclude that this technique could potentially be used as a future biomarker to detect and quantify the severity of OI, respectively (91).

### Fibrous Dysplasia

Fibrous dysplasia (FD) is a disorder in which parts of bone are replaced by fibrous connective tissue and trabecular bone of poor quality. The disease is caused by a mutations in the *GNAS1* gene and symptoms commonly present during the second decade of life. It may occur as a single lesion (monostotic) or at multiple sites of the skeleton (polyostotic). A specific polyostotic form is also known as McCune-Albright syndrome (MAS), which is accompanied by skin pigmentation and endocrinopathies. Commonly affected sites are the long bones, ribs, pelvis and the craniofacial skeleton. The *GNAS1* gene mutation leads to overproduction of cAMP in the osteoblastic cell lineage which in turn leads to increased proliferation and abnormal differentiation of osteoblastic cells. Most patients with FD are asymptomatic and FD is often an incidental finding on routine X-ray examination. In some patients, the enlargement and distortion of bone lesion(s) can cause pain, swelling, bone deformity, or pathological fractures, which are the main symptoms of FD (93). CT or MRI scans are used to determine the extensiveness of the lesion(s). For the final diagnosis histological examination is required.

A total of nine studies on FD/MAS were identified during our search (94–102). Three out of the nine studies were case-reports. In addition to standardized histomorphometry different study designs included the use of quantitative backscattered electron microscopy (qBEI), immunohistochemistry, and confocal microscopy. A lower mineral content was seen in the more severely affected FD patients measured by qBEI (94). Two immunohistochemical studies were performed to obtain a better understanding of the disease. The most remarkable findings included high expression of c-fos and c-jun in fibroblast-like cells, a negative response to nuclear antigen of cellular proliferation (PCNA), and a positive response to AgNOR (97, 102). The case report by Boyce et al. is the only study reporting the use of immunohistochemistry to evaluate the effect of denosumab treatment on the expression of RANKL. However, the biopsies before and after treatment were not of sufficient quality to compare RANKL expression (100). Two articles describing the use of confocal laser scanning microscopy report the finding of “Sharpey's fibers” in fibrous dysplastic bone (98, 99). The most recent article from 2015 by Laino et al. suggested confocal laser scanning microscopy as a useful tool to investigate FD lesions (99).

### Fibrodysplasia Ossificans Progressiva

Fibrodysplasia ossificans progressiva (FOP) is a very rare autosomal inherited disorder caused by a mutation in the *ACVR1/ALK2* gene (103). FOP is a clinical diagnosis, confirmed by genetic testing. FOP is characterized by congenital malformation of the great toes and progressive heterotopic ossification of muscles, tendons, and ligaments. Heterotopic ossification is induced by flare-ups (painful inflammatory swelling of soft tissue) (104). Nowadays diagnostic bone biopsies are not performed because of the severe risk of new heterotopic bone formation. Histological examination of bone biopsies in FOP patients has been however performed without awareness of the disease in the past. These patients lacked FOP diagnosis and genetic testing was not yet available.

Only three articles were found describing bone biopsies in FOP (105–107). Kaplan et al. performed a study during the early ‘90s to investigate bone properties by using histomorphometry and immunohistochemistry (105). Histologically, lesions with HO formation appear to be similar to original skeletal bone with regard to lamellar bone formation and resorption and marrow elements (105). In later studies by Piram et al. (106) and Ibarra et al. (107), FOP was identified by coincidence, as the acquired biopsies ruled out malignant disease.

### *PLS3* X-Linked Osteoporosis

Mutations in the actin-binding protein plastin-3 (*PLS3*) gene have recently been discovered as a rare cause of monogenetic osteoporosis which is usually reported to present without syndromic features. Due to the X-linked pattern of inheritance, homozygous male patients suffer from fractures from early childhood. Heterozygous women have a milder presentation in age of onset and fracture risk (108). DXA measurements show low BMD values in affected patients. Genetic analysis is needed for the final diagnosis. The cellular functions of PLS3 protein in bone and the pathogenic mechanism causing monogenetic osteoporosis are not well-understood. PLS3 is expressed in almost all cell types, including osteoclasts and osteocytes. It is possible that *PLS3* mutations, which affect the function or expression level of PLS3 protein in the osteocyte dendrites, lead to altered mechanosensitivity of these osteocytes.

The literature search yielded six articles, which included a bone biopsy analysis (109–114). All articles except one are case reports or case series describing phenotypical presentation of the *PLS3* mutation. Because of the disease rarity patient numbers are very low in all articles, with a maximum of five. Histomorphometric analysis showed a lamellar pattern of cortical and trabecular bone. The amount of trabecular bone is severely reduced and bone formation and resorption parameters are low. Quantitative backscattered electron microscopy showed conflicting results; the study by Fahiminiya et al. (110) described bone mineralization density distribution within the normal range, Balasubramanian et al. (114) showed a hypermineralization distribution and Kampe et al. (115) showed hypomineralization.

### Loeys-Diets Syndrome

Loeys-Diets syndrome (LDS) is an autosomal dominant disorder, due to a mutation in the *TGFBR1/2*-gene. The most typical symptoms are arterial aneurysms, hypertelorism, and bifid uvula or cleft palate. There is a wide variety of clinical presentation with involvement of other organ systems, including the skeleton. Most common skeletal findings are pectus excavatum or carinatum, joint laxity, arachnodactyly, scoliosis, and cervical spine malformation (116, 117). Also, osteoporosis with increased fracture risk has been described (118). The diagnosis of LDS is based on the variety of clinical symptoms and confirmed by genetic testing. TGF-β regulates mechanical properties and bone matrix composition (119). Dysregulation of TGF-β signaling due to the effect of the mutation in *TGFBR1/2* and its effect on bone metabolism are poorly understood.

PubMed search identified only one article. The case-series of Ben Amor et al. reported bone histomorphometric and bone material observations in two patients. Histomorphometric findings of thin cortices and high bone turnover without mineralization defects were reported. Quantitative backscattered electron microscopy was used in this case series which showed elevated matrix mineralization on the level of individual trabeculae (120).

### Osteopetrosis

Osteopetrosis is a heterogeneous group of disorders characterized by diffuse skeletal sclerosis and high bone mineral density caused by different mutations. There is a wide variety in severity of the disease ranging from asymptomatic to fatal symptoms during childhood. Most patients have an increased fracture risk due to the lack of bone flexibility. X-rays shows a dense appearance of the skeleton. Osteopetrosis is caused by decreased osteoclastic bone resorption due to failure of differentiation or function of the osteoclastic cells. The expansion of bone may cause nerve compression and hematological dysfunction due to reduction of the bone marrow cavity by central bone expansion (121, 122).

A total of six articles were identified (123–128). Histomorphometry showed multinucleated osteoclast-like cells. Four out of this, six articles described the use of transmission electronic microscopy (TEM). The most striking finding of TEM is the absence of ruffled borders and clear zones at sites of bone resorption. The article by Satomura et al. is of recent date (2007), whereas the other articles are written before the year 2000 (123, 124, 127, 128). The primary aim of all studies was focused on improved comprehension of the bone properties associated with the disease.

In addition to the stated rare metabolic bone disorders, our literature search also revealed some other rare bone disorders. Three articles described immunohistochemistry in Erdheim-Chester disease (ECD) (129–131). ECD is a disorder characterized by multi-organ infiltration of non-Langherhans' histiocytes in middle-aged patients. In almost all cases of ECD, the long bones are affected, and half of the patients have extra-skeletal involvement (132). ECD is often challenging to diagnose because of a variety of symptoms. Bone pain of the distal extremities are most often present. Histologic examination of skin or bone is required to establish the diagnosis. All the included articles were case reports or case series. It is striking that only the article by Rivera et al. described histomorphometric data (129). All three articles described findings of CD68 positive and S-100 negative histocytes. The other rare metabolic bone disorder found in the PubMed search is Cherubism. Cherubism is characterized by a bilateral swelling of the cheek caused by fibro-osseus lesions of the mandibular or maxillary bone. Age of onset is usually during childhood and sometimes the lesion regress as the child grows (133). Radiological findings shows regions of a “spongy” appearance of the maxilla and mandibular. Three articles about Cherubism described histomorphometric and immunohistochemistry data (134–136). Histopathological examination showed fibrous tissue in the lesions with multinucleated giant cells. Immunohistochemistry revealed CD68 positive multinucleated giant cells and OPG and RANKL expression.

## Discussion

This review provides an overview of the application of advanced techniques in bone biopsies of rare metabolic bone disorders and an outlook on the consideration of advanced techniques. These advanced techniques may help to resolve many gaps in the knowledge of general bone metabolism and the pathophysiology of many different rare metabolic bone disorders. Though, it has to be mentioned that histomorphometric analysis is still the most widely used method. Application of techniques focusing on structural and compositional aspects of bone could provide much more information and make a bone biopsies an even more valuable tool in diagnosis and treatment guidance. In this review, we described a variety of advanced techniques, many of which have rarely been used in clinical practice.

Clinical utility depends on several aspects of the introduction of additional technique. The information obtained from advanced techniques should refine the definition of skeletal status, predict future skeletal complications, or guide treatment in order to be of clinical value. Reference values should be available for informed decision making. The techniques should be (widely) available in diagnostic laboratories. Application of the technique should preserve its properties to allow its use in other diagnostic tools including histology/histomorphometry.

It is important to realize that some of the advanced techniques require special processing, which can hinder the usage of other methods or even make other methods impossible to use. This means that not all techniques are applicable on the same biopsy specimen. Techniques for testing mechanical properties often cause damage to the biopsy specimen by breaking it or causing surface damage. Specimen used for HPLC will be completely destroyed. Standard fixation using formaldehyde 3.5% may cause problems when immune histology assays are performed. TEM may need a different (epoxy) embedding method than acrylate for histology and HPLC may be best performed on untreated samples. Most articles combined one of the advanced analyzing techniques with histomorphometry. Only a limited amount of articles combined more than one advanced analyzing techniques in their research.

Because of the differences in processing, it will be of great importance that doctors provide the biopsy specimen with sufficient comprehensive clinical data and the specified research question to make the right choice for analyzing techniques. Starting at the bed site, a close cooperation of clinicians and researchers is needed to correctly process the biopsy for a maximum gain of information by a variety of different applied methods.

As already mentioned in the introduction of this article, most advanced analysis techniques are currently only used for research purposes only. Although some techniques are available already for many years, reference data does not always exist due to the lack of proper control groups, thus a sufficient comparison to healthy cases is lacking. This is a problem especially in the group of rare metabolic bone diseases, but also in the more common metabolic bone disorders, such as osteoporosis, where treatment-naive age-matched controls are also very limited. Reference data for adults and children have been published for bone mineral density distribution (BMDD) assessed by quantitative backscattered electron imaging (qBEI) (137, 138). Such reference data will ideally come from bone biopsies of a healthy population. Collection of these reference data will be challenging since a bone biopsy is an invasive procedure and cannot routinely be applied to healthy persons. In addition, reference data will differ between adults and children. Certainly, in (young) children ethical justification has to be considered to collect any type of reference data. A feasible option for collecting reference data from a healthy population is the use of residual material after routine surgical intervention.

Different rare metabolic bone diseases will require different methods, since they have different tissue characteristics. Rare metabolic bone disorders can roughly be divided in three groups: disorders with osteoblast dysfunction, disorders with osteoclast dysfunction, and the group of remaining disorders.

Osteoblastic dysfunction is seen in OI and fibrous dysplasia. In this group, a broad spectrum of advanced techniques could be valuable. Techniques to measure structural properties could be effective, especially in the group of OI. Confocal microscopy of polarized light microscopy could give advantageous information about the abnormal collagen fiber organization in OI or arrangement of fibrous tissue in FD. Micro-CT analysis could be of potential interest to explore the microstructural patterns compared to healthy groups. These results could provide critical insight in the interpretation of bone mineral density measurements of a DXA-scan. Also, techniques to measure material composition can potentially be of high scientific value, e.g., FTIR-spectroscopy, Raman-spectroscopy or qBEI. SAXS/WAXS technique could be of beneficial to monitor changes in mineral structure and alignment after treatment of OI patients. In OI, most of these techniques have already been tested and showed promising results. In FD, besides qBEI, none of the other techniques have been applied yet but we believe that this review will steer clinicians toward this direction, which is an effort to gain more information about the composition of the affected bone. Of course, the focal nature of the disease is a complicating factor since the bone specimen needs to be taken from an FD lesion. In OI, the mechanical properties of bone are affected as well. Testing of these properties could help the prediction of fracture risk.

Osteoclast dysfunction is seen in Paget's disorder, *PLS3* X-linked osteoporosis, and osteopetrosis. Although the affected material and mechanical parameters of bone share common characteristics, there are distinct differences. Mineral composition and structure quantifying methods such as qBEI, FTIR-spectroscopy, Raman-spectroscopy, or SAXS/WAXS may be a valuable tools to investigate the diversity of the mineral apposition, distribution, density and structure in each disease. Potentially valuable techniques are micro- and nano-indentation for the measurement of the local mechanical alteration of the bone material caused by a changed structure and composition. This has hardly been done in any of the disorder with osteoclast dysfunction. We must take into account that also the Paget's disease of bone is a focal disorder and bone samples should be obtained in the regions of interest, which are not always justifiably reachable for biopsy. *PLS3* X-linked osteoporosis is a relatively new discovery with still many uncertainties about the pathophysiological mechanism.

The remaining disorders are fibrodysplasia ossificans progressiva, Loeys-Dietz syndrome, Erdheim-Chester disease, and Cherubism. In all of these disorders, a scarce number of techniques have been tested and a lot of uncertainties about their pathophysiology exist. Analysis of structural properties and material properties will contribute to a higher level of knowledge of the disorders. In FOP, the availability of bone material for research is very low due to the rarity of the disease and the lack of biopsies or surgeries performed. Biopsies or surgery can induce heterotopic bone formation. Postmortem studies are expected to be helpful to optimize a large set of diagnostic methods and gain a deeper understanding of the disorder.

Immunological techniques have been used in the past to explore the possibility of a viral origin of some rare bone disorders. However, no such origin has been found yet. Immunohistology could be in these cases beneficial in identifying critical components of signaling and interaction between osteocytes, osteoblasts, and osteoclasts. Immunohistochemistry could also be helpful in guiding treatment especially with the new developments in biologicals. For instance, detection of RANKL by IHC in a bone biopsy of osteogenesis imperfecta, may indicate denosumab treatment could be beneficial.

The use of advanced techniques is not limited to their application in research and diagnosis; in the future they also hold great promise in the design and evaluation of treatment in patient follow-up. Firstly, techniques providing information on material properties might be able to predict fracture risk, which can be useful to differentiate the patients in need for treatment. Secondly, advanced techniques could be of great advantage to the patient and the clinician, by providing insight in the pathophysiology and giving guidance to treatment strategy. Thirdly, techniques that give insight in mechanisms by which various therapeutic drugs work, at the individual level might help in follow-up. Only a limited amount of papers discussed the use of the advanced techniques to investigate the effect of treatment. This is potentially due to the fact that two biopsies are needed, pre- and post-treatment.

Despite the development of analysis techniques for bone biopsies, innovative less invasive techniques have an obvious benefit and are thus highly sought to complement the diagnosis of metabolic bone disorders now and in the future. The measurement of biochemical bone turnover markers (BMTs) in blood serum or urine has a central role in the diagnostic process and follow-up of rare metabolic bone disorder for many years. New bone turnover markers were developed and measurements became more accurate. However, there is biological variability in BMTs due to age, sex, physical activity, recent fractures, or intercurrent disease (139). Due to these factors, interpretation of BMTs in rare metabolic bone disorders can be difficult. High-resolution peripheral quantitative computed tomography (HR-pQCT) is a new technique to detect microstructural properties at the radius and tibia (140). This non-invasive method uses a very low dose of radiation and is possible to use on a regular basis for follow-up (141, 142). Reference point indentation is a new tool to measure mechanical properties *in vivo*. Microindentations are made by a hand-held device. Although no bone biopsy is needed for this technique, patients still need local anesthetic because the indentations are painful due to penetration of the skin and periosteum (143). Both techniques are mostly used for the follow-up of more prevalent diseases such as osteoporosis; whether they are useful in the diagnosis of rare metabolic bone disorders needs further investigation.

There were several limitations in this review. The described metabolic bone diseases are rare, which could explain the low number of published articles on this topic. It could be possible that some articles were missed because the advanced analyzing techniques were not described in the title or abstract. We only used the PubMed database for data collection. The majority of the papers included descriptions of histomorphometric results. The number of patients enrolled in most of the studies was very low, especially in the papers describing techniques other than histomorphometry. Even when the number of patients in a study was sufficient, most of the time only a small number of patients underwent a bone biopsy. Also, the quality of the articles differed. One-third of the included studies consisted of case reports, with sometimes limited information. In FOP, *PLS3* X-linked osteoporosis, LD, and ECD the majority of the articles consisted of case reports. Also, 10% of the remaining articles were of poor quality (23). However, we decided to not exclude any of these articles. Given the rarity of the disorders, also these articles can help in providing an overview of performed research on bone biopsies with advanced techniques. Description of the used techniques and processing of the biopsy specimens for analysis were not always adequately.

Striking is the lack of articles where advanced techniques have been applied to study rare metabolic bone disorders with a high bone mineral density. Only a few articles describing osteopetrosis were found. Van Buchem disease was not covered in our search, an extra search for this disorder revealed no articles. Application of advanced techniques to disorders with a high bone mineral density could be valuable to accurately describe the full range of bone mineral disorders.

Notwithstanding the relatively limited amount of publications and patients, this review offers valuable insights into possible applications of various analyzing techniques.

Larger studies must establish a reliable database. Using this database, clinicians would be able to assess the need for treatment for the underlying disease as well as monitor the success of the therapy. MicroCT as well as spectroscopy and scanning electron microscopy (e.g., qBEI) are of great importance, as they link research and clinical routine. A further advantage of these techniques is the sample volume saving preparational approach. A combination of the modern techniques and classical pathological histology and histomorphometry has the potential to lead to an improved new, individualized, patient-centered therapy.

This review shows the possibilities for a broader use of bone material obtained from biopsies or residual material after surgery. These advanced techniques could provide important additional information to the new imaging techniques developed in recent years. The use of advanced analyses techniques can provide a better understanding of the pathophysiology and pave the way for new treatment options ultimately aiming optimal patient recovery and prevention of skeletal complications in the rare bone diseases.

## Data Availability Statement

All datasets generated for this study are included in the article/Supplementary Material.

## Author Contributions

Study design: ST, EE, BB, and NB. Study conduct: ST, EE, FS, BB, and NB. Data collection: ST, EE, FS, BB, and NB. Data analysis: ST, EE, FS, BB, and NB. Data interpretation: ST, EE, FS, DM, BB, and NB. Drafting manuscript: ST, EE, FS, BB, and NB. Revising manuscript content: ST, EE, FS, DM, BB, and NB. Approving final version of manuscript: ST, EE, FS, DM, BB, and NB take responsibility for the integrity of the data analysis. All authors contributed to the article and approved the submitted version.

## Conflict of Interest

The authors declare that the research was conducted in the absence of any commercial or financial relationships that could be construed as a potential conflict of interest.
